# Supplementary material for: Compensatory epistasis maintains ACE2 affinity in SARS-CoV-2 Omicron BA.1
Source: Nat Commun. 2022 Nov 16;13:7011. doi: 10.1038/s41467-022-34506-z (PMC9668218; doi:10.1038/s41467-022-34506-z)
Supplement: Supplementary file 1 — Supplementary Information [file 41467_2022_34506_MOESM1_ESM.pdf]

SUPPLEMENTARY FIGURES AND CAPTIONS

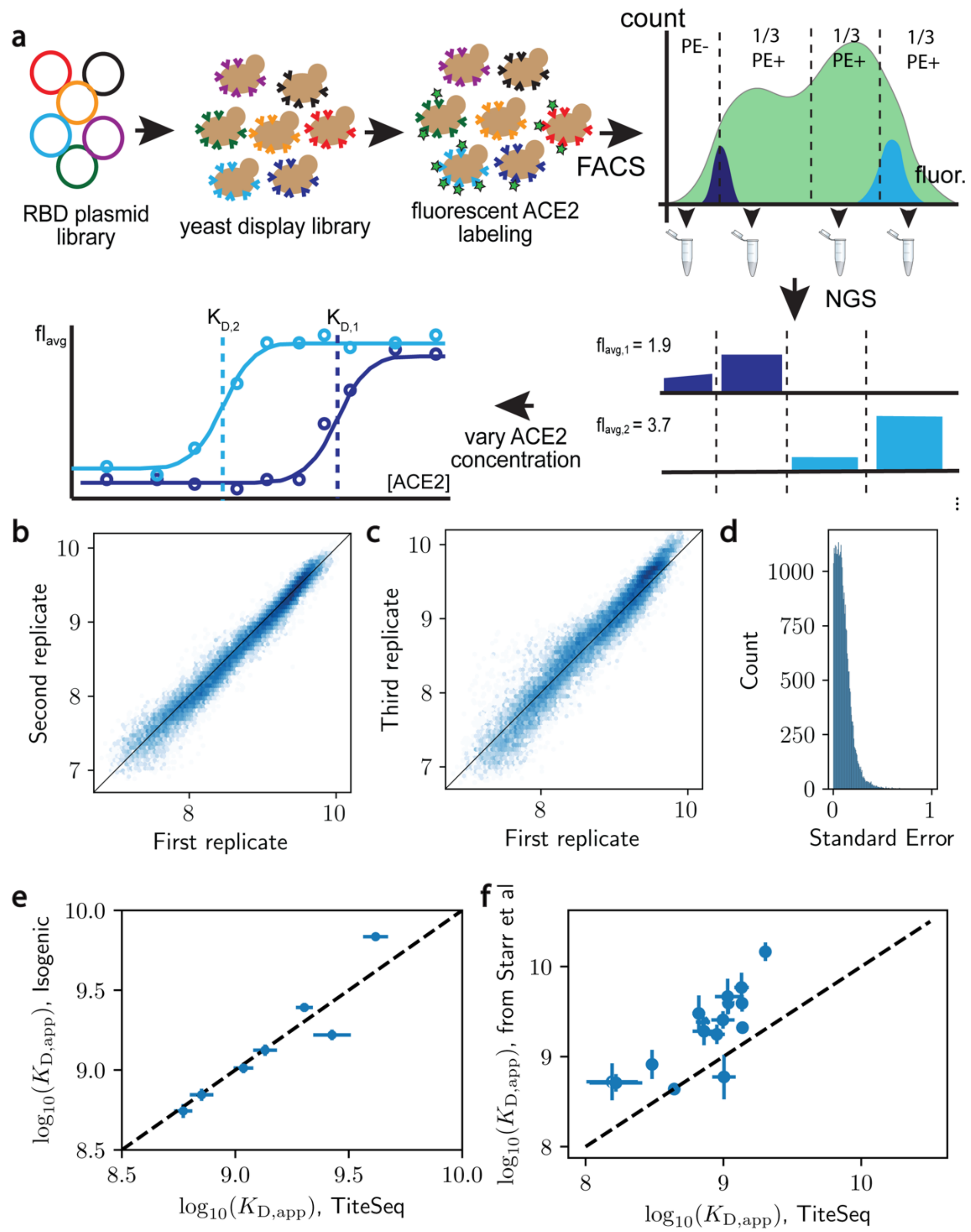

Supplementary Figure 1: Schematic overview of the experimental method and reproducibility of dissociation constants determined via Tite-seq. (a) The plasmid library of RBD variants is first transformed into a standard yeast display strain. The library is incubated with soluble, fluorescent ACE2 and sorted by flow cytometry into bins based on ACE2 fluorescence. Deep sequencing of each bin yields an estimate for the mean bin (B_{avg}) of each RBD variant. This is repeated for varying ACE2 concentration to produce a titration curve. Since the fluorescence is linearly related to the RBD occupancy on the yeast cell surface, apparent equilibrium dissociation constants can be inferred by fitting B_{avg} to the ACE2 concentration. (**b**) Correlation of -log($K_{D,app}$) between the first and second biological replicates. (**c**) Correlation of -log($K_{D,app}$) between the first and third biological replicates. (**d**) Distribution of the standard error of -log($K_{D,app}$) between biological replicates. **(e)** Isogenic measurements (see Methods) versus Tite-Seq measurement with a 1:1 dotted line. **(f)** Comparison of Tite-Seq K_D measurements with independent K_D measurements reported in Starr et al[9] with a 1:1 dotted line. Standard errors among replicates are shown by the mean-centered bars (**e,f**; n=2 vertically, n=3 horizontally).

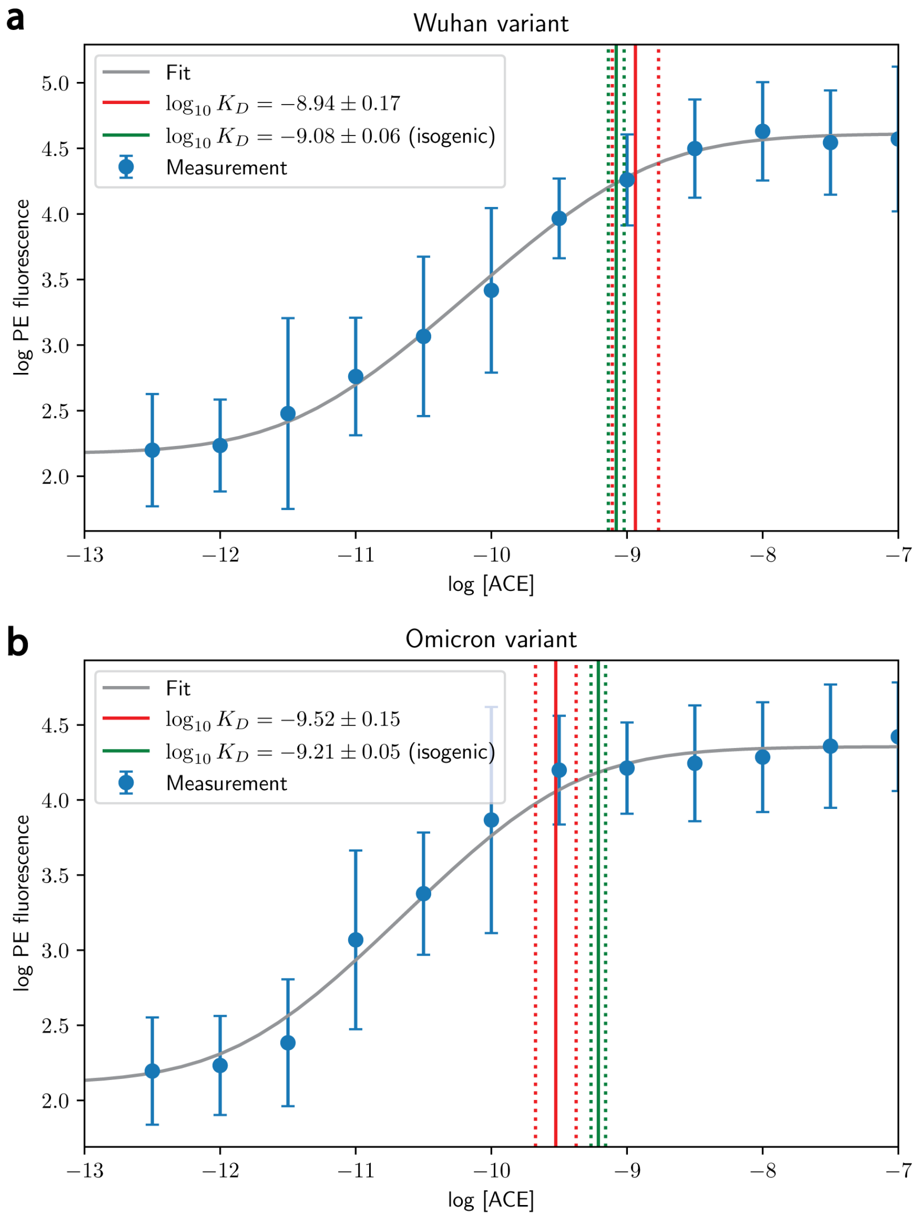

Supplementary Figure 2: Binding affinity curves of Wuhan Hu-1 and Omicron BA.1 variants. (**a**,**b**) Inferred mean log-transformed fluorescence values at each concentration (blue) for the Wuhan Hu-1 variant (**a**) and the Omicron BA.1 variant (**b**) shown across log-transformed ACE2 concentrations. The blue line represents the inferred standard deviation (centered to the mean), whereas the gray line across the concentrations is the fit of the binding curve. Inferred $K_{D,app}$ values from Tite-Seq experiment and isogenic experiment are shown by the red and green lines, respectively, and the corresponding dashed lines represent inferred errors on these values.

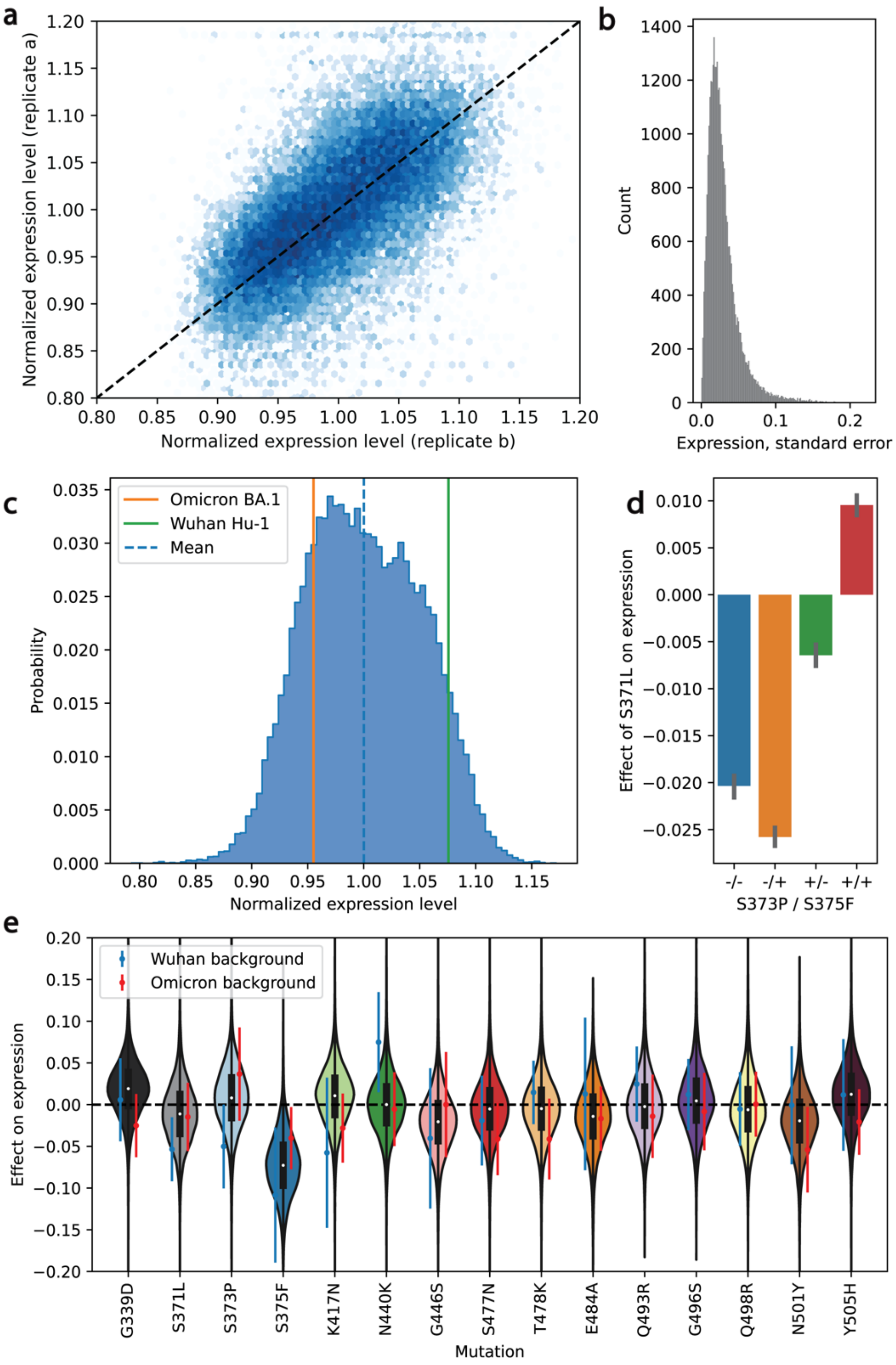

Supplementary Figure 3: Expression level of RBD in the yeast display system. (**a**) Correlation of normalized expression levels between the first and second biological replicates. (**b**) Distribution of the normalized expression levels between biological replicates. (**c**) Distribution of the normalized yeast-display expression of each RBD variant in the library. Vertical red and green lines represent the expression for Wuhan Hu-1 and Omicron BA.1, respectively. (**d**) Effect of the S371L mutation on expression levels depending on the S373P and S375F background. The error bar shows the standard error (n=4096) **(e)** Mutational effects (defined as the difference in normalized expression after adding one mutation) for each Omicron BA.1 RBD mutation. Violin plots show full distribution of effects, where black box indicate 25th and 75th percentiles and the black point denotes mean (n=16384). Blue and red points specify effects on Wuhan Hu-1 and into Omicron BA.1 variants, respectively. Error bars on these individual backgrounds represent the standard deviation (n=3)

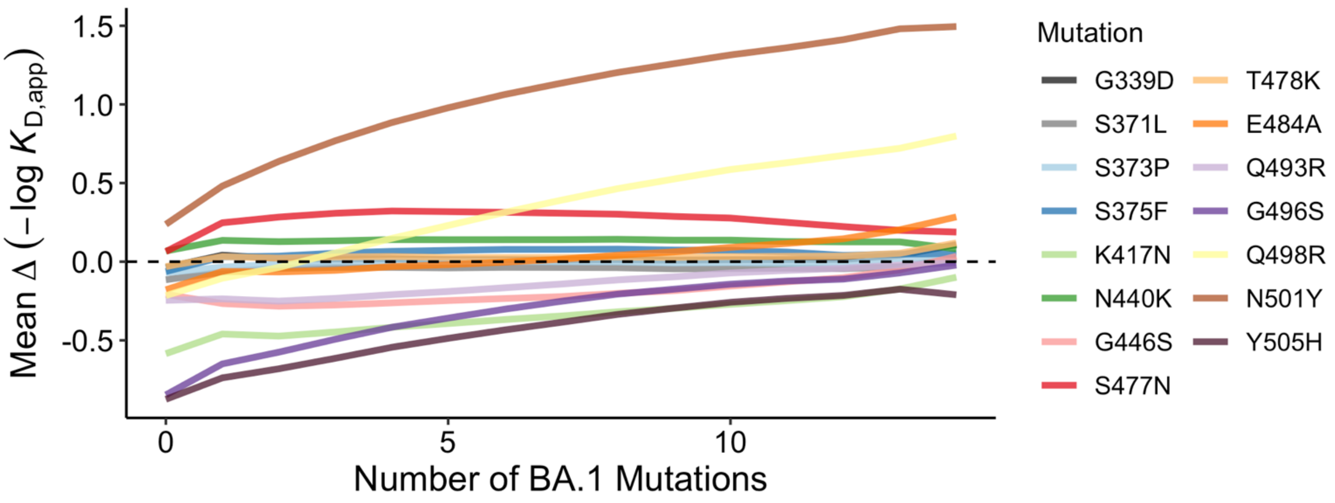

Supplementary Figure 4: Change in ACE2 affinity across number of mutations. The mean effect of each mutation is plotted against the number of BA.1 mutations in the genotypic background. Dashed line indicates no change in affinity.

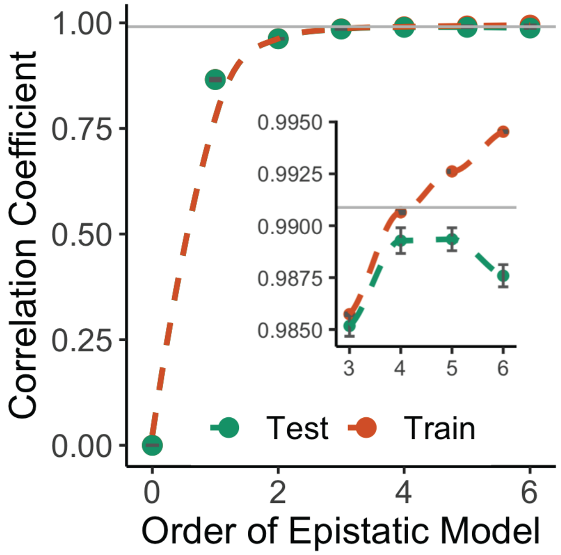

Supplementary Figure 5: Truncation of biochemical epistasis model. Correlation coefficients between the measured values of -log($K_{D,app}$) and the model estimate for various orders of epistatic model. Correlations are computed on the subset of the dataset on which the model was trained (orange) and on the hold-out subset (green), averaged over the 10 folds of cross-validation. The inset is a zoomed-in version for orders 3 to 6. Error bars represent the standard error (n=10).

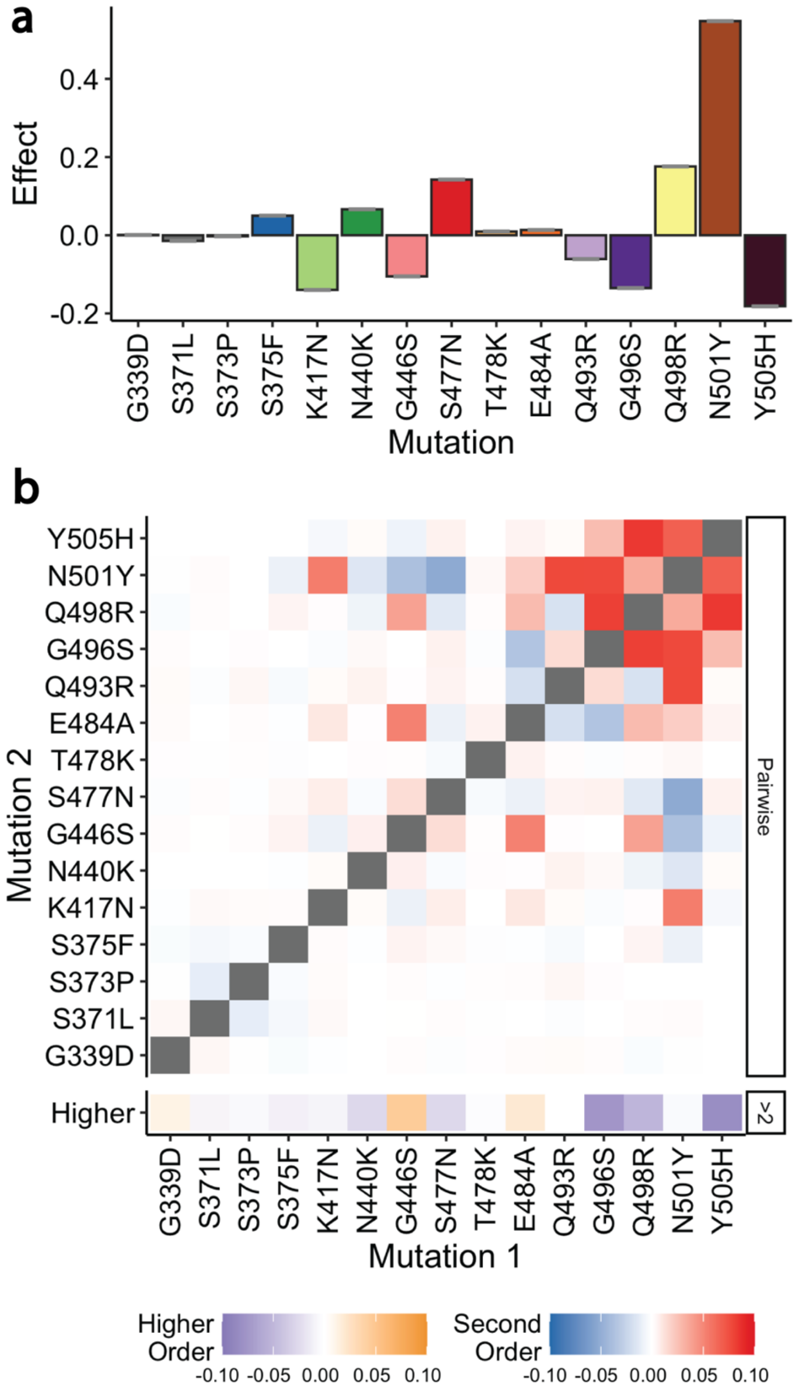

Supplementary Figure 6: Alternative model of statistical epistasis. (a) Linear effect of each mutation in the statistical epistasis model that is truncated at the fourth order. **(b)** Second-order epistatic interaction coefficients and higher order interaction in the statistical epistasis model.

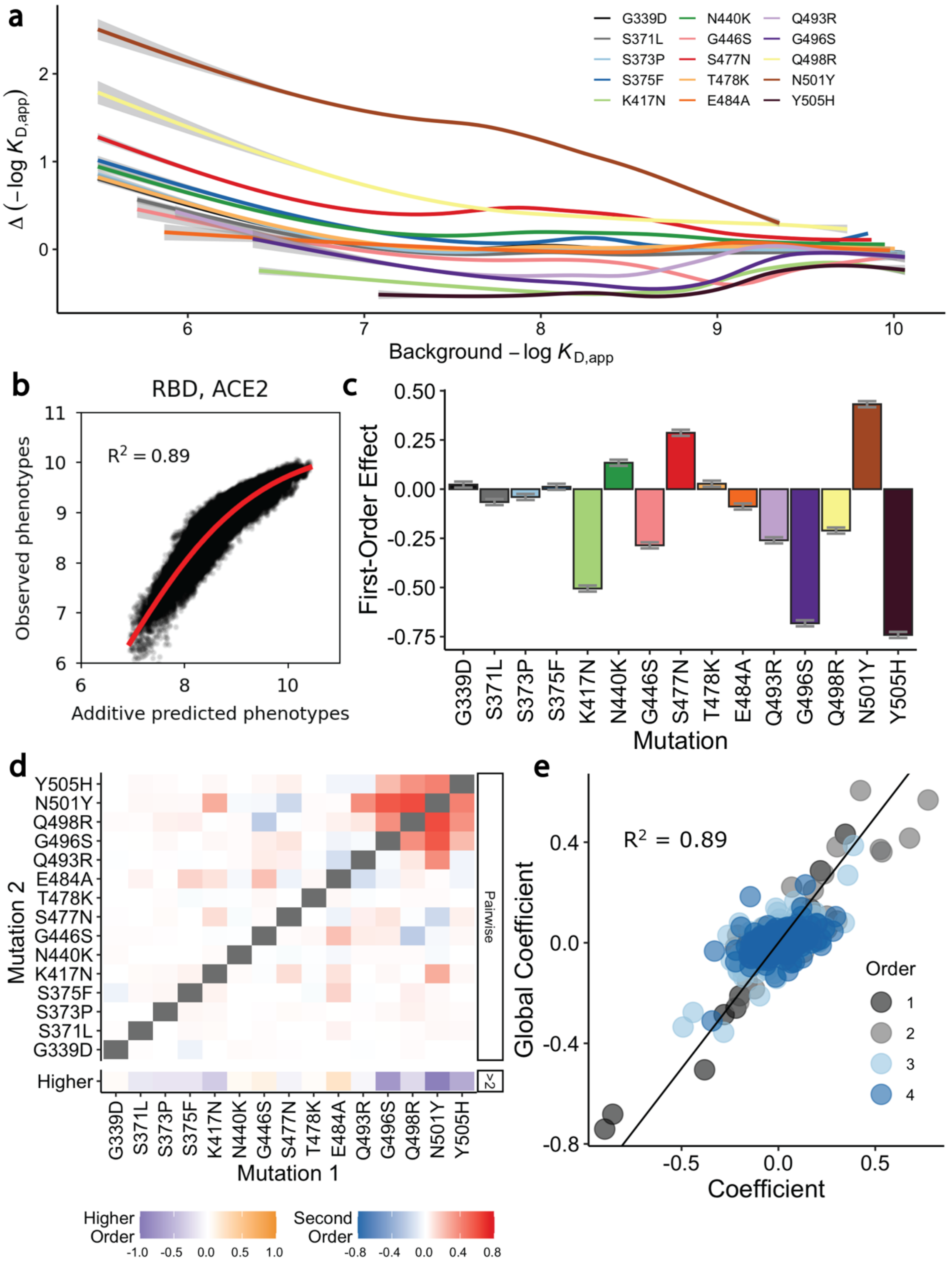

Supplementary Figure 7: Global epistasis (**a**) Relationship between the binding affinity and the mean effect of an additional mutation on this background. **(b)** Relationship between the observed binding affinity and the affinity predicted with a linear additive model without epistasis. The red line represents the global epistasis function. **(c)** Linear effect of each mutation in the global epistasis model that is truncated at the fourth order. Error bars represent the standard error (n=10). (**d**) Second-order and higher-order epistatic interaction coefficients in the global epistatic model. **(e)** Correlation between the epistatic interaction coefficients of the models with and without global epistasis. The black line represents the best fit.

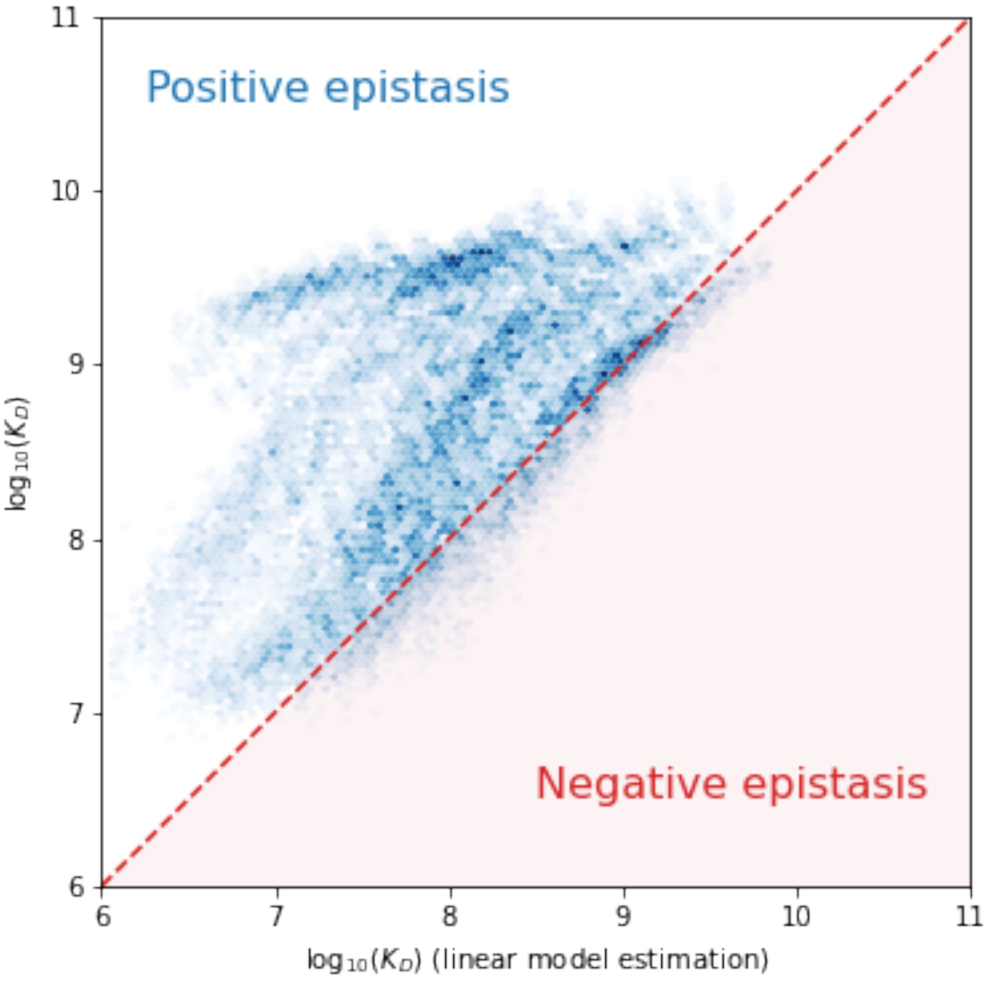

Supplementary Figure 8: Comparison between the linear model estimate of the binding affinity and the measured binding affinity. The x-axis is the predicted binding affinity, using only the linear coefficients of the full 5th-order model; the y-axis is the measured binding affinity.

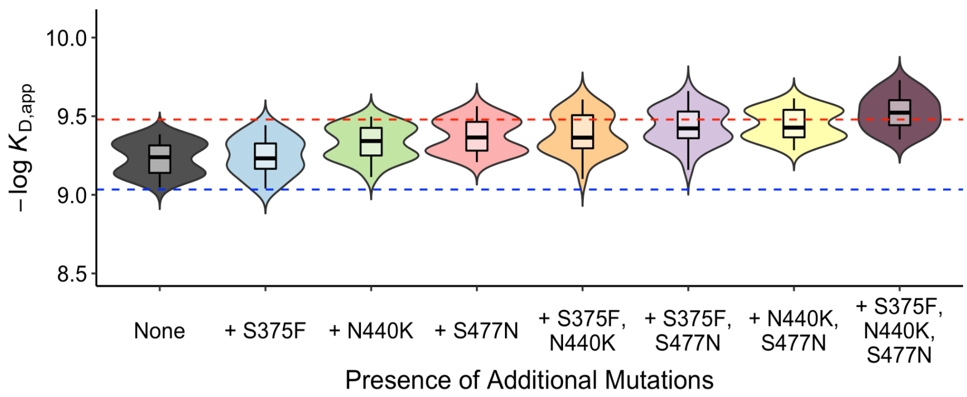

Supplementary Figure 9: Binding affinity of escape genotypes with additional compensatory mutations. The ACE2 binding affinities of variants with all seven mutations discussed in the main text (the five escape mutations K417N, G446S, E484A, Q493R, and G496S, plus Q498R and N501Y) with all possible combinations of three other mutations (S375F, N440K, and S477N). Blue and red dashed lines represent Wuhan Hu-1 and Omicron BA.1 affinity, respectively. Box bounds represent the 25th and 75th percentiles respectively.

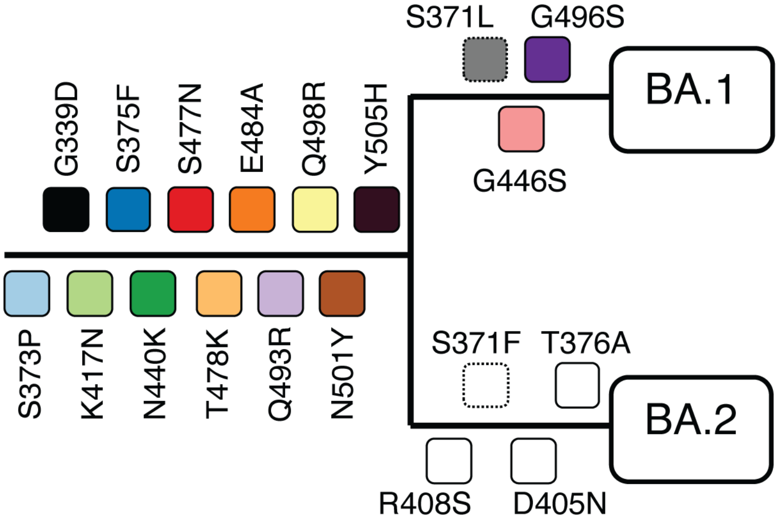

Supplementary Figure 10: Phylogeny of BA.1 and BA.2 showing mutations in spike protein RBD. Mutations are colored as in Figure 2A. Dashed boxes indicate mutations with ambiguous positions on the tree.

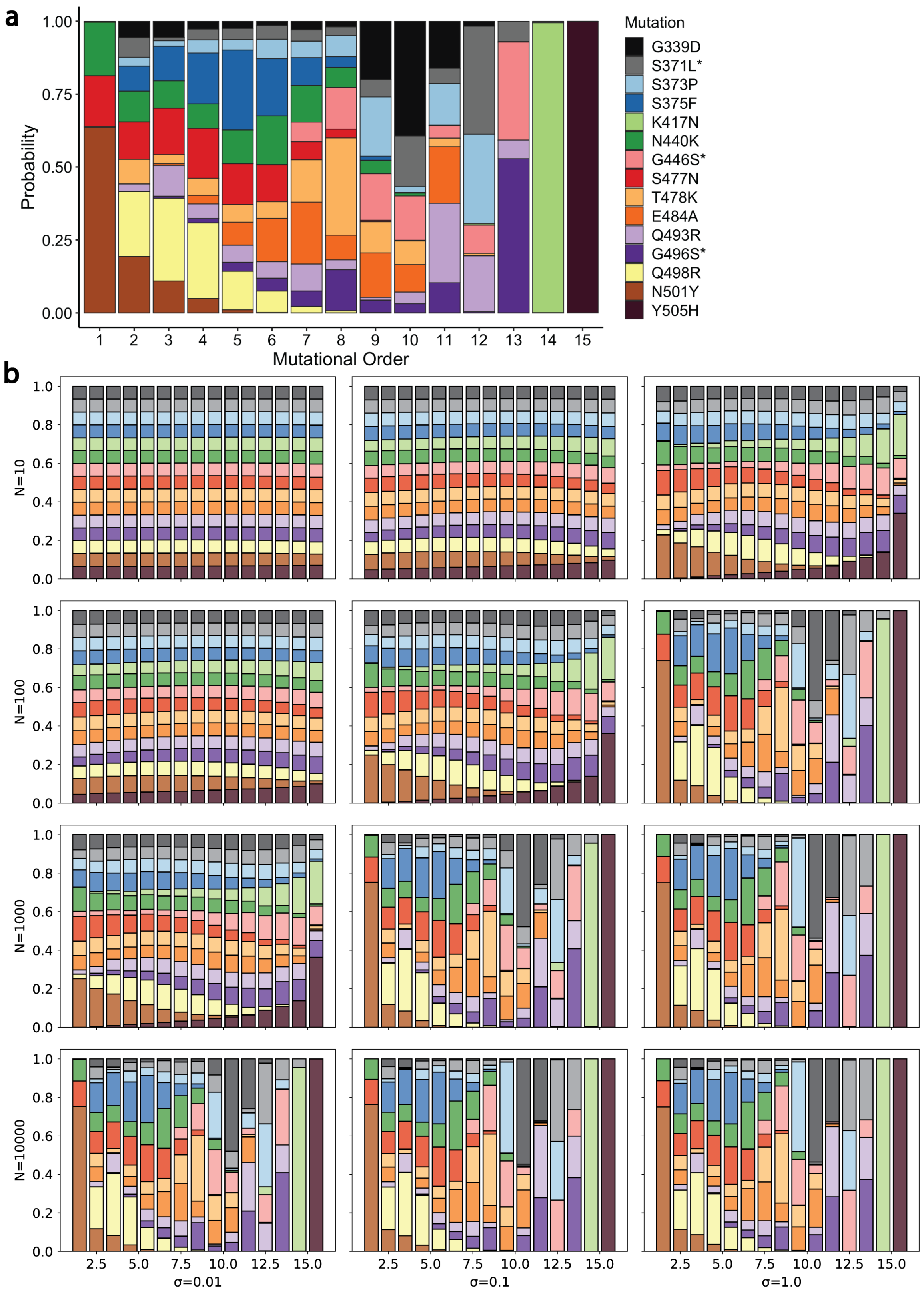

64 **Supplementary Figure 11: Inferred order of mutations.**
65 (**a,b**) Conditional probability of mutation order with N=100 and sigma = 1 (**a**) and varying N and sigma (**b**)
66 from Wuhan-Hu-1 to Omicron BA.1 variant, assuming a classical population dynamics model (see
67 Methods). Mutations with asterisks are known to happen last (see Supplementary Figure 10).
