# Supplementary material for: Compensatory epistasis maintains ACE2 affinity in SARS-CoV-2 Omicron BA.1
Source: Nat Commun. 2022 Nov 16;13:7011. doi: 10.1038/s41467-022-34506-z (PMC9668218; doi:10.1038/s41467-022-34506-z)
Supplement: Supplementary file 2 — Description of Additional Supplementary Files [file 41467_2022_34506_MOESM2_ESM.pdf]

File Name: Supplementary Data 1
Description: All coefficient values from the biochemical epistasis model (truncated at the fifth order). Performance corresponds to the R^2 of the fit, and params denotes the number of parameters inferred. Each row includes the mutation identities, coefficient value, p-value (two-tailed) of the coefficient, and its confidence intervals.

File Name: Supplementary Data 2
Description: Sequence map of the gblock that was inserted into the expression plasmid. The sequence is codon optimized (see Methods) and excludes all BsaI sites.

File Name: Supplementary Data 3
Description: Sequence map of the ccdB that was inserted into the expression plasmid for the Golden Gate Assembly during library construction.

File Name: Supplementary Data 4
Description: Primer sequences used to generate clonal strains ('gibson_primers_gblocks_incorp'), golden gate fragments ('gg_primers'), ccdB plasmid ('ccdb_primers'). Fragment sequences ordered for Golden Gate Assembly are also included ('fragment').

File Name: Supplementary Data 5
Description: All primer sequences used in the two rounds of PCR ('round_1' and 'round_2') to generate the sequencing library.
