# Supplementary material for: Compensatory epistasis maintains ACE2 affinity in SARS-CoV-2 Omicron BA.1
Source: Nat Commun. 2022 Nov 16;13:7011. doi: 10.1038/s41467-022-34506-z (PMC9668218; doi:10.1038/s41467-022-34506-z)
Supplement: Supplementary file 3 — Supplementary Data 1–5 [file 41467_2022_34506_MOESM3_ESM.zip › supplementary_files/Description of Additional Supplementary Files.pdf]

File Name: Supplementary_Data_1-ACE2_5order_biochem_coefficients.txt
Description: All coefficient values from the biochemical epistasis model (truncated at the fifth order). Performance corresponds to the R^2 of the fit, and params denotes the number of parameters inferred. Each row includes the mutation identities, coefficient value, p-value (two-tailed) of the coefficient, and its confidence intervals.

File Name: Supplementary_Data_2-gblock_omicron_TWIST_BsaI_removed.dna
Description: Sequence map of the gblock that was inserted into the expression plasmid. The sequence is codon optimized (see Methods) and excludes all BsaI sites.

File Name: Supplementary_Data_3-gblock_ccdB_gene_up_down_Twist.dna
Description: Sequence map of the ccdB that was inserted into the expression plasmid for the Golden Gate Assembly during library construction.

File Name: Supplementary_Data_4-golden_gate_primers_and_fragments.xlsx
Description: Primer sequences used to generate clonal strains ('gibson_primers_gblocks_incorp'), golden gate fragments ('gg_primers'), ccdB plasmid ('ccdb_primers'). Fragment sequences ordered for Golden Gate Assembly are also included ('fragment').

File Name: Supplementary_Data_5-illumina_library_sequencing_primers.xlsx
Description: All primer sequences used in the two rounds of PCR ('round_1' and 'round_2') to generate the sequencing library.
